# The role of DNA damage response in amyotrophic lateral sclerosis

**DOI:** 10.1042/EBC20200002

**Published:** 2020-10-20

**Authors:** Yu Sun, Annabel J. Curle, Arshad M. Haider, Gabriel Balmus

**Affiliations:** 1 UK Dementia Research Institute at University of Cambridge, Cambridge CB2 0AH, U.K.; 2 Department of Clinical Neurosciences, University of Cambridge, Cambridge CB2 0AH, U.K.

**Keywords:** amyotrophic lateral sclerosis, DNA damage response, Frontotemporal dementia, Genome instability, Neurodegeneration, Repeat expansion

## Abstract

Amyotrophic lateral sclerosis (ALS) is a rapidly disabling and fatal neurodegenerative disease. Due to insufficient disease-modifying treatments, there is an unmet and urgent need for elucidating disease mechanisms that occur early and represent common triggers in both familial and sporadic ALS. Emerging evidence suggests that impaired DNA damage response contributes to age-related somatic accumulation of genomic instability and can trigger or accelerate ALS pathological manifestations. In this review, we summarize and discuss recent studies indicating a direct link between DNA damage response and ALS. Further mechanistic understanding of the role genomic instability is playing in ALS disease pathophysiology will be critical for discovering new therapeutic avenues.

Amyotrophic lateral sclerosis (ALS) is a lethal degenerative motor neuron disease with a median survival of 2–4 years after diagnosis [1] and no available effective treatment [2]. Caused by loss of motor neurons in the motor cortex, brain stem and spinal cord, the worldwide annual incidence of ALS is approximately 1 per 50,000 live births and is expected to exponentially increase in the next 20 years [3]. Since it leads to severe disability with high fatality rate, there is an extensive socioeconomic burden alongside the unmet medical need [1,4,5]. Most likely, as for most neurodegenerative diseases, one of the reasons for the slow progression in the development of novel therapies in ALS is the fact that the underlying neurodegeneration may start decades before clinical diagnosis [6–10]. Thus, a better understanding of the disease mechanisms that appear early and represent common triggers in both familial (fALS) and sporadic (sALS) forms of ALS is required as to inform on early diagnostic/prognostic markers and therapies.

Although ALS is a mainly sporadic disease (90–95% of patients) [11], attention has been focused on the 5–10% of patients that have fALS where a gene-disease association can be made (Figure 1). Currently around 30 genes associated with fALS have been identified [12–16] and while a common molecular mechanism remains uncertain, recent evidence suggests that accumulation of genomic instability (GIN) – via impaired DNA damage recognition or defective DNA repair – is one of the hallmarks of ALS (Figure 2 and Table 1) [17].

**Figure 1:**
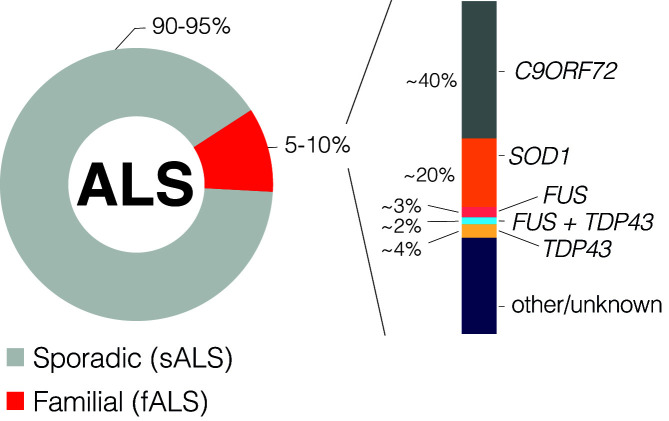
ALS patient stratification Although some genetic heterogeneity is observed across the world, literature suggests these are the approximate proportions of ALS patients with mutations in the represented genes. Table 1 highlights other genetic contributors and their links to DDR.

**Figure 2:**
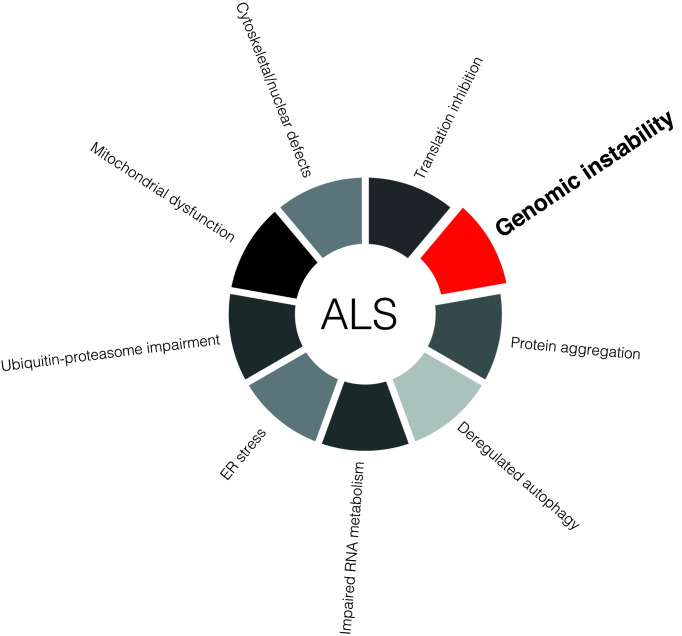
Molecular hallmarks of ALS Current evidence suggests several underlying etiological factors in ALS. Genomic instability, caused by defective DNA damage signalling or DNA repair, toxic DNA repair, impaired clearance of endogenous genotoxic stressors (i.e. ROS), or due to imbalanced chromatin structure states, could be a unifying pathophysiological characteristic of the disease.

**Table 1:** DDR associated mutations in ALS

Gene	DDR link
***TDP-43***	*TAR DNA-binding protein 43*; ALS-linked mutations [145]; impairs DDR in ALS [34]
***FUS/TLS***	*Fused in sarcoma*; ALS-linked mutation [79,146]; impairs DDR in ALS [147]
***HNRNP***	*Heterogeneous nuclear ribonucleoprotein*; modifies TDP43 [148,149]; associated with DDR [150]; hnRNP L recruits 53BP1 and BRCA1 in cancer [151]; hnRNP F,H, and K are related to ALS [152] and p53 recruitment [153]
***HNRNPA1***	ALS linked mutations [154]; telomere protection and telomerase activation [155]; regulated by TDP43 [156]
***HNRNPA2/B1***	ALS linked mutations [154]
***SARM1***	*Sterile alpha and TIR motif containing 1*; ALS linked mutations [157]; *SARM1* deletion suppresses TDP43-linked ALS [158]
***PFN1***	*Profilin-1*; mutated PFN1 aggregates and shifts TDP43 from nuclei to cytoplasm in ALS [159]
***UBQLN2***	*Ubiquilin-2*; ALS-linked mutations [160]; interacts with TDP43 [161]
***CCNF***	*Cyclin F*; ALS-linked mutations [162]; increases ubiquitinated TDP43 [162]
***ERBB4***	*Erb-B2 Receptor Tyrosine Kinase 4*; interacts with TDP43 [163]; regulates p53-dependent DDR [164]; interacts with KAP1 for DDR [165]; activates p53 and p21 [166]
***SIGMAR1***	*Sigma nonopioid intracellular receptor 1*; interacts with TDP43 [167]
***GLE1***	RNA export mediator; ALS-linked mutations [168]; interacts with TDP43 [169]; *GLE1* deletion increases phosphorylated H2AX, decreases BRCA1 and FANCD2 and increases ATR resulting in delayed DDR [170]
***SOD1***	*Superoxide dismutase*; ALS-linked mutations [33]; protects DNA from oxidative stress damage in ALS [171]
***DAO***	*D-amino acid oxidase*; ROS production [172]
***KIAA1563/ALS2***	*Alsin*; ALS-linked mutations [154]; increases ROS in ALS [173]; regulates autophagy [174]
***C9ORF72***	Induces DNA damage in ALS [175]
***SETX***	*Senataxin*; encodes a DNA/RNA helicase protein involved in DDR and RNA production in ALS4 (Juvenile ALS) [176,177]
***ATXN2***	*Ataxin-2*; ALS-linked mutations [178]; R-loop suppressor [65]; affects R-loop in ALS [179]
***VCP***	*Valosin-containing protein*; ALS-linked mutations [180]; facilitates 53BP1 recruitment for DSB repair [17,181]; causes p62 accumulation in ALS [182]
***NEK1***	*NIMA-Related Kinase 1*; mutation induces DNA damage in ALS and impairs ATM-mediated DDR [183]
***C21ORF2***	NEK1 interactor; involved in HR repair [48,157]
***MATR3***	*Matrin-3*; activated by ATM and involved in the early stage of the DSB response [184]
***SQSTM1 /p62***	*Sequestosome-1*; inhibits nuclear RNF168; an E3 ligase essential for histone H2A ubiquitination and DDR [185]
***TBK1***	*TANK-binding kinase 1*; ALS-linked mutations [157]; an inducer of type-1 interferons; major role in autophagy and mitophagy [186]; cGAS/Sting/TBK1/IRF3 regulates p21 maintaining chromosomal stability [138]
***ELP3***	*Elongator complex protein 3*; ALS-linked mutations [154]; binds to PCNA; linked to DNA replication and repair [187]
***TIA1***	*T-cell intracellular antigen 1*; affects DDR; binds to p53 mRNA and controls p53 expression [188]; promotes phase separation and alters SG dynamics in ALS [15]

Without excluding the importance and relevance of other molecular mechanisms that have been extensively covered by others [18–21], in this review we will examine the evidence revealing a role for the DNA damage response (DDR) in ALS, by discussing some of the particular genes, proteins and cellular processes implicated at the intersection of the DDR and ALS.

## Sources of genomic instability and connection to ALS

DDR is starting to be recognized as a unifying mechanism in neurodegenerative disorders [22]. DNA damage can arise from both endogenous and exogenous sources and if not repaired will lead to the accumulation of GIN and ensuing pathologies [23]. To enable normal neuronal functions and survival, the DDR encompasses complex mechanisms that recognize DNA damage and signal for DNA damage repair [22,24]. Increasing evidence indicates that mature neurons are highly dependent on accurate DDR and that DNA damage accumulation accelerates in the normal human brain particularly after 40 years of age [25]. With ageing, there is thus an even greater requirement for DDR, and failure to deal with GIN accumulation will eventually lead to increased neuronal loss. Paradoxically, the DDR is known to change and deteriorate with age [26]. GIN arises from the buildup of lesions, such as base modifications, abasic sites, single- or double-stranded breaks (SSBs; DSBs) [27,28]. DSBs are particularly deleterious and, if left unrepaired, are detrimental to cell survival. DSBs are repaired by homologous recombination (HR) and non-homologous end joining (NHEJ). Carried out exclusively in cells that are in S- or G2-phase of the cell cycle, HR is the preferential DSB repair pathway as it is a relatively error-free process. Because under physiological conditions neurons are outside of the replicative cell cycle in the quiescent G0-phase, even though error-prone, NHEJ is the primary repair pathway for DSBs. That being said, recent evidence suggests that in addition to classical NHEJ, neurons could employ transcription-coupled repair mechanisms utilizing mRNA as a template for homology directed repair [29].

Since mature neurons are post-mitotic non-replicating cells that are difficult to replace [30,31], unsanctioned neuronal loss will lead to neurodegeneration. Concomitantly, ageing also brings other imbalances that can accelerate such DDR-related processes [32].

In ALS, the endogenous sources contributing to deleterious accumulation of GIN are from both impaired removal of reactive metabolic genotoxins (i.e. reactive oxygen species; ROS) that can overwhelm DDR [33] and from the incapability to recognize or repair DNA damage [34,35]. Although in this review, we are focusing on endogenous sources of DNA damage, one must keep in mind the geographical heterogeneity of ALS that cannot be explained by genetic risk factors alone [12,36]. Thus, future research should consider environmental genotoxic influences that might also play a role in both sALS and fALS.

## SOD1 and DNA damage in ALS

Superoxide dismutase 1 (SOD1) is a free radical scavenging enzyme that in the cytoplasm catalyzes the conversion of superoxide anions formed during mitochondrial respiration into hydrogen peroxide [37] and protects motor neurons – which are particularly prone to the toxic effects of mutant and dysfunctional SOD1 – against oxidative damage and neurodegeneration [33]. In both sALS and fALS, SOD1-induced neuronal toxicity occurs through gain-of-function mutations (Figure 1) [33,38] that lead to accumulation of injuries produced from the unscheduled free radical attack on pyrimidine and purine bases [39,40]. Secondarily, in both sALS and fALS, SOD1 can be secreted as monomers into the extracellular space leading to cell death [41]. Unexpectedly, recent data show that independent from its catalytic function, SOD1 performs additional roles in the nucleus. In response to elevated ROS, in an ataxia-telangiectasia mutated (*ATM*; a core DDR gene [42]) dependent manner, CDP-diacylglycerol synthase 1 (CDS1) kinase phosphorylates SOD1 at S60 and S99 promoting rapid SOD1 translocation to the nucleus where it regulates the expression of a large set of genes involved in oxidative stress defence and DDR [43]. Furthermore, nuclear SOD1 increases *SpeedyA1* (*SPY1*) expression promoting cell survival and inhibiting damage-induced apoptosis. In ALS, pathologic SOD1–G93A cannot translocate to the nucleus and exercise its protective role via *SPY1* regulation [44]. SPY1 is a nuclear protein that controls the transition between G1- and S-phases of the cell cycle via checkpoint-dependent kinase 2 (CDK2) activation [45]. In neurons, re-entry into cell cycle (CCR) is partly controlled by ATM, is atypical, and leads to neuronal death [30,46,47]. The observation that SOD1 can influence such decisions will require further investigation especially since other fALS genes, such as *NEK1, C21ORF2* and *CCNF* are also involved in cell cycle progression [48–51], suggesting CCR should be considered in ALS pathology.

**Figure 3:**
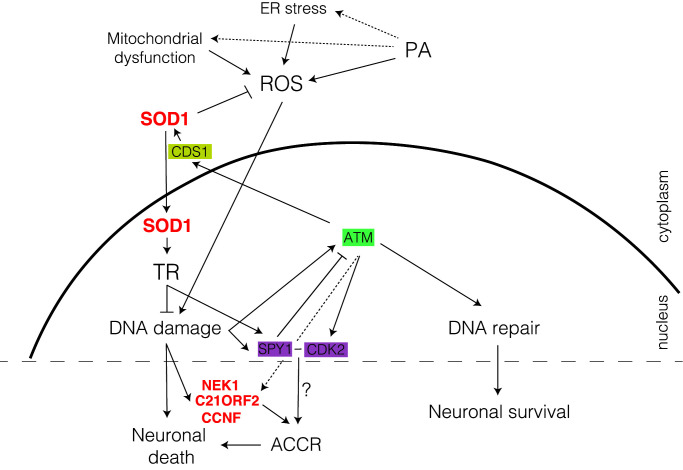
SOD1 plays a dual role in DDR SOD1 nuclear translocation is ATM/CDS1-dependent. Once SOD1 enters the nucleus, it activates transcription (TR) of many genes that are involved in DDR or ROS defence. SOD1 regulates *SPY1* expression, which activates CDK2, a G1- to S-phases check point. Other cell cycle regulation (CCR) gene mutations (e.g. *NEK1, C21ORF2* and *CCNF*) are implicated in defective DDR, suggesting a role for atypical cell cycle re-entry (ACCR) in ALS. Mutated genes identified in ALS (red), homologous recombination (HR; green), atypical cell cycle checkpoint (AACR; purple) and ROS regulation (yellow). Dotted arrows are proposed, yet not completely proven, interactions.

Thus, SOD1 protection against DNA damage accumulation is bi-modal, with the first tier of defence being executed in the cytoplasm through ROS scavenging, and the second in the nucleus where it controls the expression of DDR-related genes (Figure 3).

## TDP43 mislocalization impairs DDR

**Figure 4:**
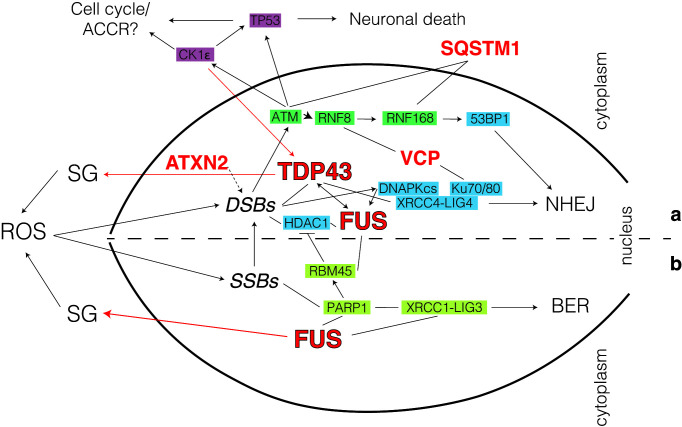
Role for TDP43 and FUS in maintaining genome stability in ALS Pathway choice is directed by the balance between TDP43 and FUS interaction at break sites. Simplified model for the role of TDP43 (**a**) and FUS (**b**) in DDR. Mutated genes identified in ALS (red), atypical cell cycle checkpoint (AACR; purple), non-homologous end-joining (NHEJ; blue), homologous recombination (HR; green) and base excision repair (BER; chartreuse). Dotted arrows are proposed, yet not completely proven, interactions.

Transactivation response DNA-binding protein 43 (TDP43) is a highly conserved nuclear protein that acts as transcription and splicing regulator as well as scaffold for nuclear bodies [52]. While in normal conditions TDP43 is primarily localized in the nucleus, in disease states it gets trapped in insoluble cytoplasmic inclusions (stress granules; SG; see Figure 4a) [53,54]. Although mutated TDP43 accounts for only approximately 4% of fALS cases (Figure 1), TDP43-SG accumulation is a pathology characteristic for ∼95% of all ALS and ∼50% of frontotemporal dementia (FTD) cases [21,55–57], as well as a secondary pathology in other neurodegenerative diseases, including Alzheimer’s [58], Parkinson’s [59] and Huntington’s diseases [60,61].

Pathologic TDP43 mislocalization activates the mitochondrial unfolded protein response [62], elevates ROS levels and affects cytoplasmic-nuclear trafficking, eventually leading to increased neuronal stress and subsequent cell death [63,64]. Associated with such stress, GIN accumulation was described in sALS and fALS patients as well as in model organisms with orthologous TDP43 loss-of-function [34]. In addition, TDP43 cytoplasmic retention can be aggravated by other factors such as ataxin 2 (ATXN2), itself associated with DDR processes [65], thereby further increasing the risk of developing ALS [66]. Furthermore, TDP43 mislocalization and GIN accumulation maintain a vicious cycle via casein kinase 1ε (CK1ε) that has been shown to promote cytoplasmic accumulation of TDP43 [67]. Together with other CK1 isoforms, CK1ε is activated upon GIN build-up and controls several cellular processes linked to DNA damage signalling and repair, including apoptosis and cell cycle checkpoint control (Figure 4a) [68].

Although initially the connection between TDP43 dysfunction and the accumulation of GIN in ALS was thought to be a secondary feature, recent evidence shows that neuronal TDP43 plays an important direct role in DDR by controlling the nuclear recruitment of the XRCC4-DNA ligase 4 (LIG4) complex, critical for DSB repair via NHEJ [63]. In ALS/FTD, TDP43 nuclear exclusion incapacitates the transport of XRCC4/LIG4 leading to abortive NHEJ with consequent accumulation of toxic DSBs. The involvement of DSB repair in ALS/FTD is further substantiated by the observation that other proteins mutated in fALS such as valosin-containing protein (VCP)/p97 and sequestosome 1(SQSTM1)/p62 are linked to NHEJ [69,70]. VCP has been shown to directly interact with the canonical NHEJ proteins Ku70/80 [69] as well as with ring finger proteins (RNF) 8/168 [71] to balance DNA repair pathway choice and promote cell survival. This process is done in close correlation with SQSTM1/p62 that via interactions with ATM, RAD50 and RNF168 also regulates the choice between HR and NHEJ in favour of the latter [70]. The TDP43/XRCC4 direct connection is somewhat unexpected as replicating cells have less of a requirement for TDP43 in NHEJ (Figure 4a). This should prompt a more detailed analysis of these pathways in neurons where the relationship between different DDR components might be rewired. Further studies will be required to look, for example, at the interplay between TDP43 and other NHEJ proteins, such as PAXX and XRCC4-like factor (XLF) [72] or the SHIELDIN complex [73]. Additionally, given its RNA-binding capabilities, TDP43 has been implicated in impeding DNA:RNA hybrids (R-loops) formation [17,74]. This places TDP43 squarely in the middle of both DSB repair and the transcriptional stress that neurons endure.

## FUS-mediated solid-to-liquid phase transition promotes DDR in ALS

Fused in sarcoma (FUS) is a nuclear ribonucleoprotein involved in a variety of cellular functions including transcription, protein translation and RNA splicing and transport [75,76]. Initially studied for its roles in cancer [77,78], it was later discovered that around 5% of fALS and 1% of sALS cases are associated with *FUS* mutations (Figure 1) [79].

Following oxidative damage, in a poly(ADP-ribose) polymerase (PARP1)-dependent manner, FUS facilitates the recruitment of XRCC1/LIG3 to SSBs and enhances LIG3 ligation activity thus promoting base excision repair (BER; Figure 4b) [80–83]. These interactions are, at least, partly based on the ability of FUS to rapidly traffic to the nucleus, as mutations in the nuclear localization sequence induce FUS aggregation, genomic instability, and consecutive neurodegeneration [84]. Additionally, in ATM and DNA-PKcs-dependent manner, FUS is involved in DSB repair by directly controlling the recruitment of histone deacetylases 1 (HDAC1) to chromatin [35]. The involvement of FUS in HDAC1 recruitment and activation is bimodal. Firstly, following DSB induction, FUS recruitment of HDAC1 promotes deacetylation and activation of NHEJ [85]. Secondly, in a PARP-dependent manner, FUS interacts with RNA-binding motif protein 45 (RBM45) and prevents excessive recruitment of HDAC1 [86].

These data build a model in which FUS controls the choice between SSB repair and DSB repair pathways in healthy neurons. Further research will be required to specifically understand the connection between FUS and TDP43 in DDR as well as the requirement of HR versus microhomology mediated end-joining (alternative NHEJ; MMEJ). In some patients, ALS is evidenced to manifest on the basis of oligogenic rather than monogenic alterations, with summative effects from several DDR pathologies [87], as indicated in Figure 4.

## C9ORF72 repeat expansion and impaired DDR in ALS

Nucleotide repeat expansion (NRE) disorders encompass more than 20 human genetic diseases, most of which affect the nervous system, that arise from an expansion of a particularly unstable tandem of 3–12 DNA bases [88,89]. The deleterious effects of these NREs depend on the location of the repeat within the affected gene, its sequence, as well as the size of the repeat. *C9ORF72* ALS/FTD is caused by the expansion of a hexanucleotide GGGGCC (G4C2) track in the first intron of the *C9ORF72* gene [90].

**Figure 5:**
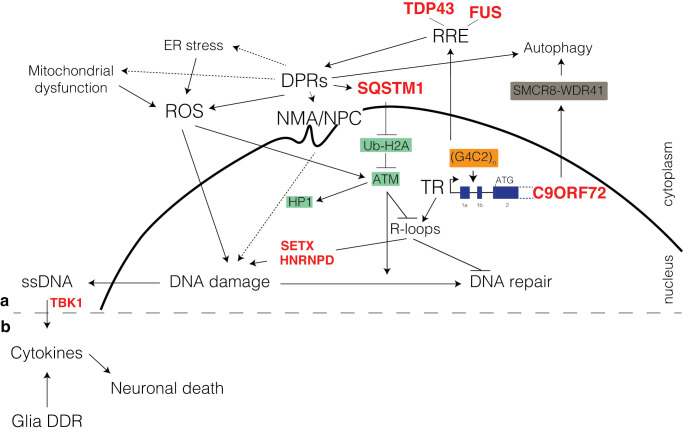
DDR defects in ALS with C9orf72 mutations G4C2–NREs in the first intron of the *C9ORF72* gene increases RRE, which impairs DDR through binding to RNA-binding proteins. Transcription over G4C2–NREs leads to R-loop formation and subsequent DNA damage accumulation. RAN translation produced DPRs that can increase ROS, induce nuclear membrane alterations (NMA) and may potentially sequester DDR proteins. NMA include structural and functional disturbances at the nuclear pore complexes (NPC) involving transport receptors. Abnormal nucleo-cytoplasmic transport of both RNA and proteins at NPC has been suggested to be, either related to molecule sequestrations by DPR and RRE or in parallel with other factors, a strong *C9ORF72* disease modifier. G4C2–NREs also decreases *C9ORF72* expression, which impairs autophagy and exacerbates DPRs accumulation. Mutated genes identified in ALS (red), homologous recombination (HR; green) and autophagy (brown). Dotted arrows are proposed, yet not completely proven, interactions.

G4C2–NREs are pathogenic through several non‐exclusive mechanisms that can all influence the accumulation of DNA damage lesions and affect their repair (Figure 5A). Initially, transcription over G4C2 tracks is problematic and will lead to accumulation of R-loops [91,92] and accumulation of toxic DNA secondary structures, hairpins and G-quadruplexes, which require DDR to be resolved [93,94]. Intriguingly, mutations in the R-loop processing factors *senataxin* (*SETX)* and *HNRNPD* also lead to fALS [95,96]. Subsequently or in parallel, transcripts containing repeats can form RNA repeat expansion (RRE) foci that will bind and sequester various RNA‐binding proteins such as TDP43, FUS, nucleophosmin (NPM1) or AP endonuclease (APE1), potentially altering their localization and DDR functions [91,97–105]. Finally, the G4C2–NREs are non‐AUG (RAN) translated into dipeptide repeats (DPR)‐containing proteins (poly-GR; -GP; -GA; -PR; -PA) that form inclusions throughout the brain of patients with ALS/FTD [106–109] and can lead to endoplasmic reticulum (ER) stress, mitochondrial dysfunction with ROS accumulation [110] and sequestration of DDR proteins [111,112]. Moreover, DPRs can accumulate at the nuclear membrane and the nuclear pore complex (NPC) to promote nuclear membrane abnormalities (NMA), impaired nuclear-cytoplasmic transport [113–115] and imbalanced chromatin states [116]. Furthermore, in a vicious feedback loop, the expanded G4C2 can interfere with the transcription and translation of the C9ORF72 mRNA thus leading to decreased autophagy and further accumulation of DPRs [117].

Consequence of these pathologic mechanisms, C9ORF72 ALS/FTD patients show increased GIN accumulation both in the brain [118] and spinal cord [97] where presence of DDR markers can be detected. One of the clearest evidences for a direct DDR deficiency in ALS comes from the observation that expressing RREs and/or DPRs results in elevated R-loop levels and DSBs build-up in rat neurons, human cells and C9ORF72 ALS patient spinal cord tissues. This is as a result of the incapability of C9ORF72-ALS neurons to mount a suitable DDR signalling cascade which occurs due to defective ATM-mediated signalling that arises as a consequence of SQSTM1/p62 accumulation and impaired H2A ubiquitylation (Figure 5A). Most likely due to this ATM signalling problem, NHEJ seems to be up-regulated to toxic levels that can be rescued in fly models via Ku (NHEJ), APEX1 (BER) or ERCC1 (interstrand cross-link DNA repair) dysregulation [119]. Although more information is needed to understand where the NHEJ or other DNA repair dependent toxicity is coming from, such observation would be in line with similar mechanisms present in ATM deficient replicating cells [120].

Another important link between G4C2 expansion and DDR is the observation that DPR accumulation leads to imbalanced chromatin states with impact on DNA repair [121]. Poly-PR, for example, specifically binds DNA at heterochromatin, evicts HP1α and causes abnormal histone H3 methylation leading to altered chromatin structure and NMA [116]. In response to endogenous DNA damage, to activate DDR, HP1α is phosphorylated by ATM [122], while H3K9me3 is required to activate the acetyltransferase activity of TIP60 [123]. Moreover, DPR accumulation at the nuclear membrane can lead to nuclear membrane rupture with subsequent GIN [124,125] as well as bi-directional transport defects at the NPC resulting in impaired shuttling of RNA and proteins. Such transport disturbances might interfere with factors involved in DDR and DNA repair, further feeding a vicious circle of DNA damage with insufficient repair [57,84,126]. These mechanisms might also influence the onset of age-related ALS, as perturbed nucleo-cytoplasmic cargo delivery is itself a feature of the CNS ageing process [127]. Thus, because the NPC has been shown to play important roles in DNA repair and the organization of genome architecture [128] while in response to DNA damage chromatin undergoes dramatic genome-wide changes that are at the heart of DDR [129], further scrutiny will be required to apprehend the relationship between nuclear DPR accumulation at specific nuclear structures (i.e. NPC), imbalanced chromatin states and their link to DDR in ALS.

## Neuroinflammation and DDR in ALS

It must be highlighted that neurons do not live in isolation, and neurodegeneration is associated with microglial reactivity and activation of innate immune responses. Neuroinflammation is a common characteristic of ALS and comprises the stimulation of microglia, astrocytes and inflammatory T cells [130]. Upon activation, these cells secrete proinflammatory cytokines, such as tumour necrosis factor α, interferon γ, and interleukin 1β [131,132]. Typically, the innate immune response is activated by the presence of foreign cytoplasmic DNA via activation of cyclic GMP–AMP synthetase, (cGAS), and the cyclic dinucleotide receptor, stimulator of interferon genes (STING) [133]. Recently, more attention is being given to the link between accumulating GIN, the subsequent leakage of DNA in the cytoplasm and the activation of the cGAS-STING cascade [134]. In this model, ALS-GIN accumulating neurons can amass increasing amounts of single-stranded DNA (ssDNA) in the cytoplasm and promote neuroinflammation with production of cytokines and subsequent neuronal death (Figure 5B). Interestingly, haploinsufficiency in the STING activating kinase TANK-binding kinase (TBK1) [135] is associated with fALS and FTD [136,137] (Figure 5B). Within this pathway, TBK1 is important for several functions, including maintenance of chromosomal stability [138]. A functional cGAS/STING pathway is also known to be required for normal chromosomal segregation in cancer cells via a p21-dependent mechanism modulating G2/M transition [138]. The putative genome surveillance role in post-mitotic non-replicating cells is less clear.

Neuroinflammation with subsequent neurodegeneration can also result from a glia autonomous problem in dealing with DDR. Mutant human TDP43 expressed specifically in Drosophila glial cells causes DNA damage, elevated replication of retrotransposable elements (RTE) [139], and Gypsy endogenous retroviruses [140] and apoptosis in the nearby neurons. During their replication, the expression of RTE cDNA can lead to genome instability and accumulation of DSBs [141]. These studies highlight that TDP43 mutations in glial cells promote ALS progression, at least partly through impaired DDR signalling. Among glia, aberrant astrocyte function has also been implicated in ALS pathology which has been discussed extensively by others and merits further research [142–144]. Further studies will be required to better understand the relationship between DDR and neuroinflammation in ALS/FTD.

## Conclusion

ALS is one of the most common adult-onset neurodegenerative disorders. Currently, ALS is fatal and incurable with patients expected to survive ∼2–4 years after diagnosis, revealing an urgent need for effective therapeutic strategies. Proof of DNA damage accumulation and DNA repair deficiency in both ALS initiation and progression is amassing, highlighting the fact that genomic instability is a hallmark of disease pathogenesis. Shedding light on the specific DDR mechanisms at play has important therapeutic potential.

## Summary

- Genomic instability is a hallmark of both sporadic and familial ALS with many ALS genes involved in recognition or repair of DNA damage.
- Outside of the nucleus SOD1 works to impede ROS accumulation and in the nucleus to influence DNA damage response via transcription regulation.
- TDP43 and FUS work mainly to balance the pathway choice between SSB repair and DSB repair.
- Expansion of a repeated G4C2 track in the *C9ORF72* gene leads to impaired ATM signalling.
- Genomic instability may be a starting point for neuroinflammation in ALS.

## Open Access

Open access for this article was enabled by the participation of University of Cambridge in an all-inclusive *Read & Publish* pilot with Portland Press and the Biochemical Society under a transformative agreement with JISC.

## Abbreviations

ALS: amyotrophic lateral sclerosis
APE1: AP endonuclease 1
ATM: ataxia-telangiectasia mutated
ATXN2: Ataxin 2
BER: base excision repair
CCR: cell cycle re-entry
CDK2: checkpoint-dependent kinase 2
CDS1: CSP-diacylglycerol synthase 1
CK1ε: casein kinase 1ε
DDR: DNA damage response
DRP: dipeptide-repeat proteins
DSB: DNA double-strand break
ER: endoplasmic reticulum
fALS: familial ALS
FTD: frontotemporal dementia
FUS: fused in sarcoma
G4C2: hexanucleotide GGGGCC
GIN: genomic instability
HDAC1: histone deacetylases 1
HNRNPD: heterogeneous nuclear ribonucleoprotein D
HR: homologous recombination
LIG4: ligase 4
MMEJ: microhomology mediated end-joining
NEK1: NIMA-related kinase 1
NHEJ: nonhomologous end-joining
NMA: nuclear membrane abnormalities
NPM1: Nucleophosmin 1
NRE: nucleotide repeat expansion
PARP1: Poly (ADP-ribose) polymerase 1
RAN: Non‐AUG; AUG is a start codon
RBM45: RNA-binding motif protein 45
RNF: Ring finger protein
ROS: reactive oxygen species
RRE: RNA repeat expansion
RTE: retrotransposable elements
sALS: Sporadic ALS
SETX: Senataxin
SG: stress granules
SOD1: superoxide dismutase 1
SPY1: SpeedyA1
SQSTM1: Sequestosome 1
SSB: single-stranded break
ssDNA: single-standed DNA
STING: stimulator of interferon genes
TBK1: TANK-binding kinase 1
TDP43: transactivation response DNA-binding protein 43
VCP: Valosin-containing protein
XLF: XRCC4-like factor
